# Size control and vacuum-ultraviolet fluorescence of nanosized KMgF_3_ single crystals prepared using femtosecond laser pulses

**DOI:** 10.1080/14686996.2016.1241659

**Published:** 2016-10-19

**Authors:** Sotaro Muramatsu, Masahiro Yanagihara, Toru Asaka, Shingo Ono, Tomohito Nagami, Kentaro Fukuda, Toshihisa Suyama, Yuui Yokota, Takayuki Yanagida, Akira Yoshikawa

**Affiliations:** ^a^ Nagoya Institute of Technology, Nagoya, Japan; ^b^ Tokuyama Corporation, Tokyo, Japan; ^c^ Institute for Materials Research, Tohoku University, Sendai, Japan; ^d^ Nara Institute of Science and Technology, Nara, Japan

**Keywords:** Fluoride, VUV, nanoparticles, pulsed laser ablation, cathodoluminescence, 40 Optical, magnetic and electronic device materials, 102 Porous / Nanoporous / Nanostructured materials, 204 Optics / Optical applications, 305 Plasma / Laser processing, 503 TEM, STEM, SEM, 505 Optical / Molecular spectroscopy

## Abstract

We fabricated nanosized KMgF_3_ single crystals via a dry pulsed laser ablation process using femtosecond laser pulses. The sizes, shapes, and crystallographic properties of the crystals were evaluated by transmission electron microscopy (TEM). Almost all of the particles were spherical with diameters of less than 100 nm, and they were not highly agglomerated. Selected-area electron diffraction and high-resolution TEM analyses showed that the particles were single crystals. Particle diameter was controlled within a wide range by adjusting the Ar ambient gas pressure. Under low gas pressures (1 and 10 Pa), relatively small particles (primarily 10 nm or less) were observed with a high number density. With increasing pressure, the mean diameter increased and the number density drastically decreased. Vacuum-ultraviolet cathodoluminescence was observed at 140–230 nm with blue shift and broadening of spectrum.

## Graphical abstract

**Figure:**
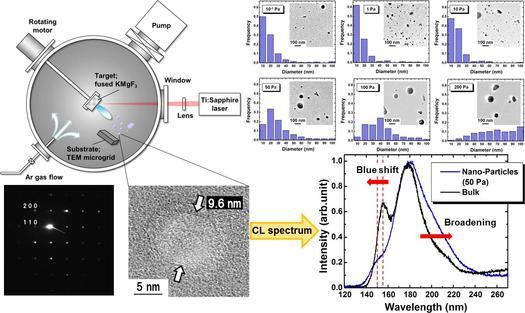

## 1. Introduction

Ternary metal fluorides such as A^I^M^II^F_3_ (A = alkaline metal elements; M = bivalent metal elements) have received considerable attention as important inorganic functional materials. They have interesting physical and chemical properties such as ferromagnetism, nonmagnetic insulator behavior, and piezoelectric characteristics along with advantageous optical properties.[1,2]

For example, ternary KF compounds such as KMgF_3_ and KCaF_3_ have been studied as scintillators.[3,4] They show fast fluorescence in the vacuum-ultraviolet (VUV) region because of the electronic transitions from the F^−^ 2p valence band to the K^+^ 3p core band (i.e. crossluminescence).

KMgF_3_ has excellent characteristics such as homogeneous optical properties, high thermal stability, anisotropy, high optical transparency, and high luminous efficiency.[5–7] Rare earth ion-doped KMgF_3_ has been widely studied for use in dosimeters, laser rods, and scintillators.[8–10] KMgF_3_ was also applied as a VUV phosphor in our recent study wherein we grew a KMgF_3_ thin film phosphor via pulsed laser deposition and developed a field emission VUV lamp using the KMgF_3_ thin film.[11] The lamp operated in the wavelength range of 140–220 nm, which is the shortest wavelength ever reported for a solid-state phosphor lamp.

Owing to these promising results, it is important to expand this area of research to the nanometer scale. In the past few years, the preparation and characterization of materials on the nanometer scale has provided not only new physics in reduced dimensions, but also the possibility of fabricating novel materials. Particularly in luminescent materials, many researchers have investigated semiconductors based on materials such as Si, SiC, CdSe, and ZnO.[12–24] These studies revealed that quantum effects result in nanoparticles having different luminescence properties in terms of quantum efficiency, luminescent decay, and emission wavelength.[25,26] The most interesting result is that the emission shifts to a shorter wavelength with decreasing particle size. For example, with decreasing particle diameter, Si nanoparticles show a blue shift in luminescence from 900 nm (average particle diameter = 4.8 nm) to 610 nm (average particle diameter = 2.8 nm).[12] This attractive phenomenon must also be present in other multiple phosphors; however, there are only a few reports on fluoride composite materials, and their synthetic routes are limited to liquid-phase methods such as thermal decomposition.[5,27–31]

In this study, we fabricated KMgF_3_ nanoparticles via a dry process using pulsed laser ablation (PLA). The synthesis of nanocrystals via PLA was recently reported [22,32]; this preparation method has the distinct advantage of high cleanliness because the high-energy laser beam can be focused on a small spot on the surface of the target in the presence of a high-purity inert gas. Thus, PLA is a non-contact process, and contamination from a crucible is avoided.[33,34] In this study, we also applied femtosecond laser pulses as such pulses were previously shown to reduce the differences in the compositional ratio from the source target and particles above 100 nm.[35,36] In addition, it is important to investigate the luminescence properties of nanosized KMgF_3_ in the VUV region because luminescence in this region is important in applications such as surface treatment, optical cleaning of semiconductor substrates, and sterilization. Although a solid-state phosphor is required as a substitute for the gas phosphor, there are few existing solid-state phosphors that possess high quantum efficiency in the VUV region. Thus, improving the luminescence properties and particularly changing the emission wavelength by atomization would greatly enhance the applications of VUV light sources.

## 2. Experimental methods

**Figure 1.**
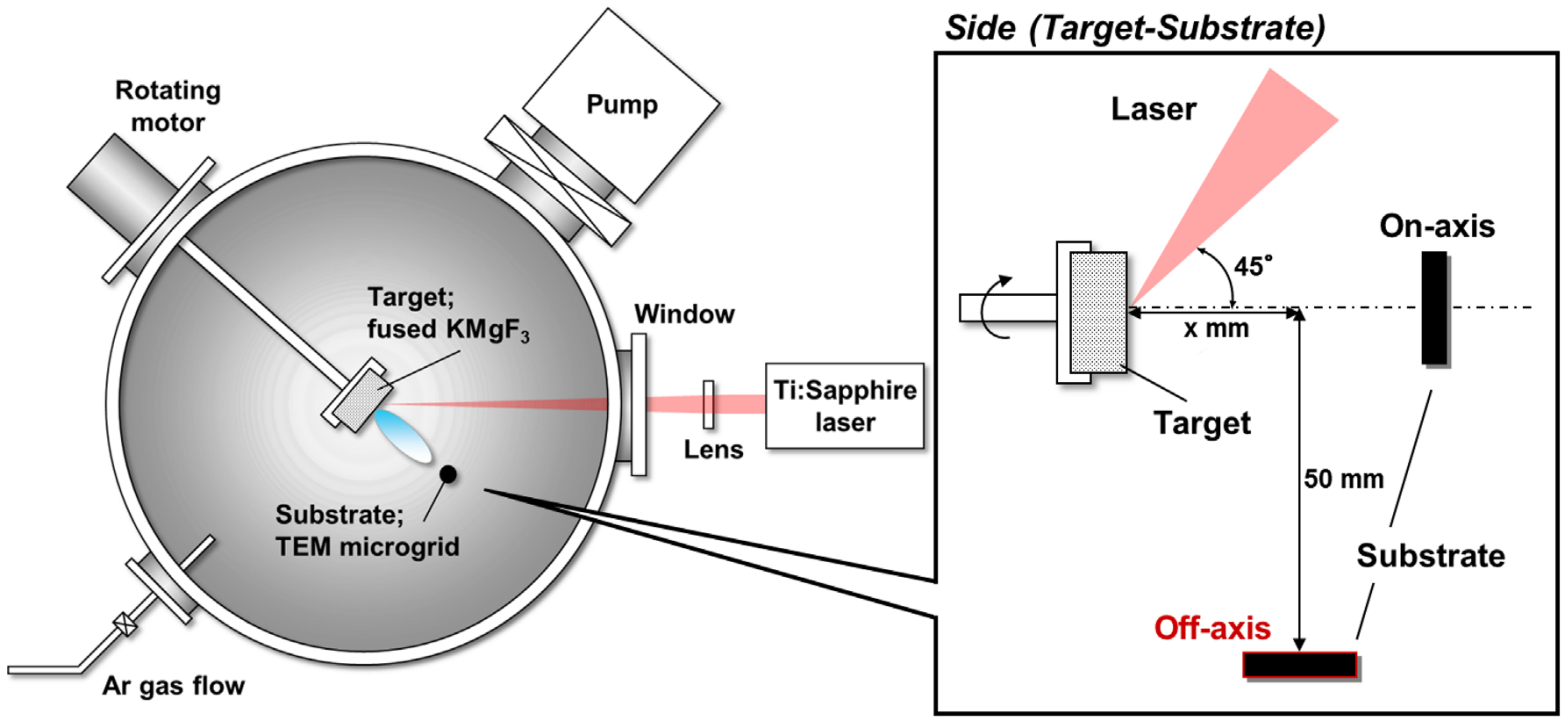
Experimental setup for the fabrication of KMgF_3_ nanoparticles.

Figure 1 shows a schematic drawing of the experimental setup. The fused KMgF_3_ target (KF:MgF_2_ = 1:1) was placed in a vacuum chamber with a base pressure of 10^−1^ Pa and prepared by melting under Ar:CF_4_ (95:5) atmosphere at 1220°C. After melting, the target took 1 h to reach 900°C and an additional 48 h to reach room temperature. The target was then irradiated with femtosecond laser pulses at a wavelength of 790 nm, a pulse width of 206 fs, and a repetition rate of 1 kHz. The laser beam was focused on the target with a fluence of 11 J cm^–2^. The particles were collected on a transmission electron microscopy (TEM) microgrid. For thin film fabrication via PLA, the substrate was generally placed toward the target (on-axial position). In contrast, we placed the TEM microgrid in an off-axial position (*x* = 25 mm, 50 mm below the target) to prevent the deposition of large particles. For irradiation, the chamber gate valve connected to the pumping system was closed, and ambient Ar gas was leaked into the chamber. Experimental studies have been conducted on the effects of ambient gas pressure on the sizes and shapes of nanoparticles.[37,38] Thus, in this study, the Ar gas pressure was adjusted as the dominant parameter within the range of 10^−1^ to 200 Pa to investigate its influence on particle size.

## 3. Results and discussion

**Figure 2.**
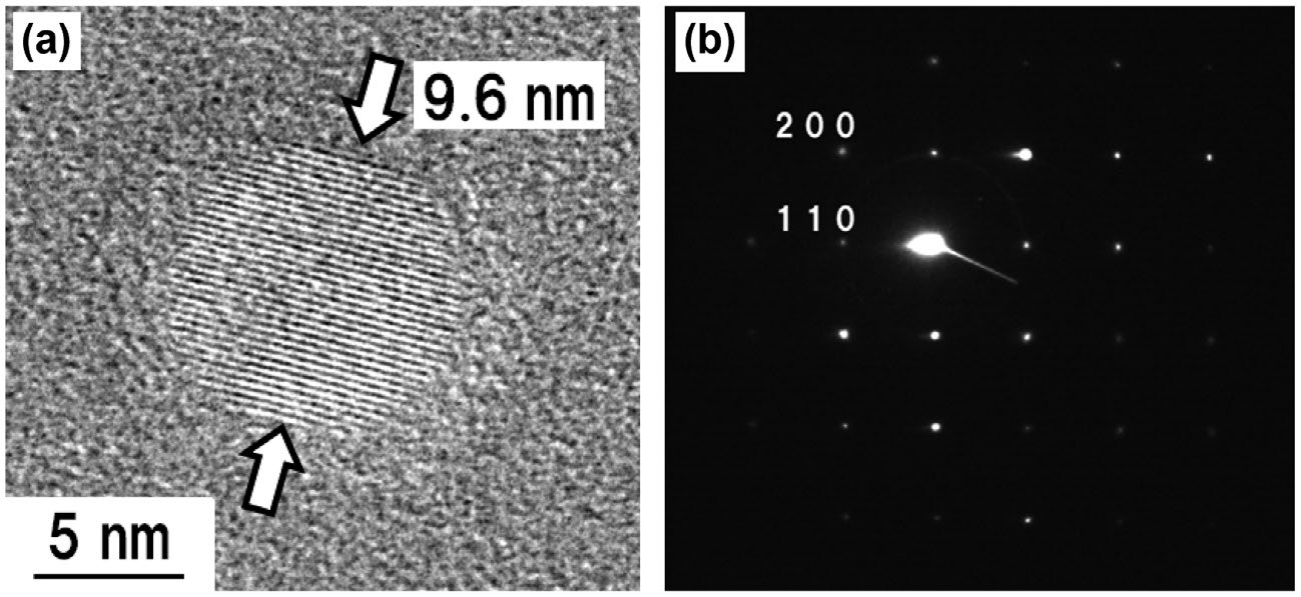
(a) HR-TEM image and (b) SAED pattern of a KMgF_3_ nanoparticle prepared at an Ar pressure of 50 Pa.

The crystallographic properties of the fabricated KMgF_3_ nanoparticles were investigated by high-resolution TEM (HR-TEM) and selected-area electron diffraction (SAED; Figure 2). Figure 2(a), which was obtained by observing a KMgF_3_ nanoparticle prepared at 50 Pa at a magnification of 300,000 × , clearly shows the crystal lattice fringes. Figure 2(b) shows narrow diffraction spots from a KMgF_3_ nanoparticle that was prepared under an Ar pressure of 50 Pa. This pattern indicates the single crystallinity of the sample. The prominent diffractions of the (200) and (110) planes in the SAED pattern are consistent with cubic KMgF_3_. Approximately equivalent results were obtained for the particles fabricated at other Ar pressures. These results indicate that nanosized KMgF_3_ single crystals were successfully fabricated by PLA.

**Figure 3.**
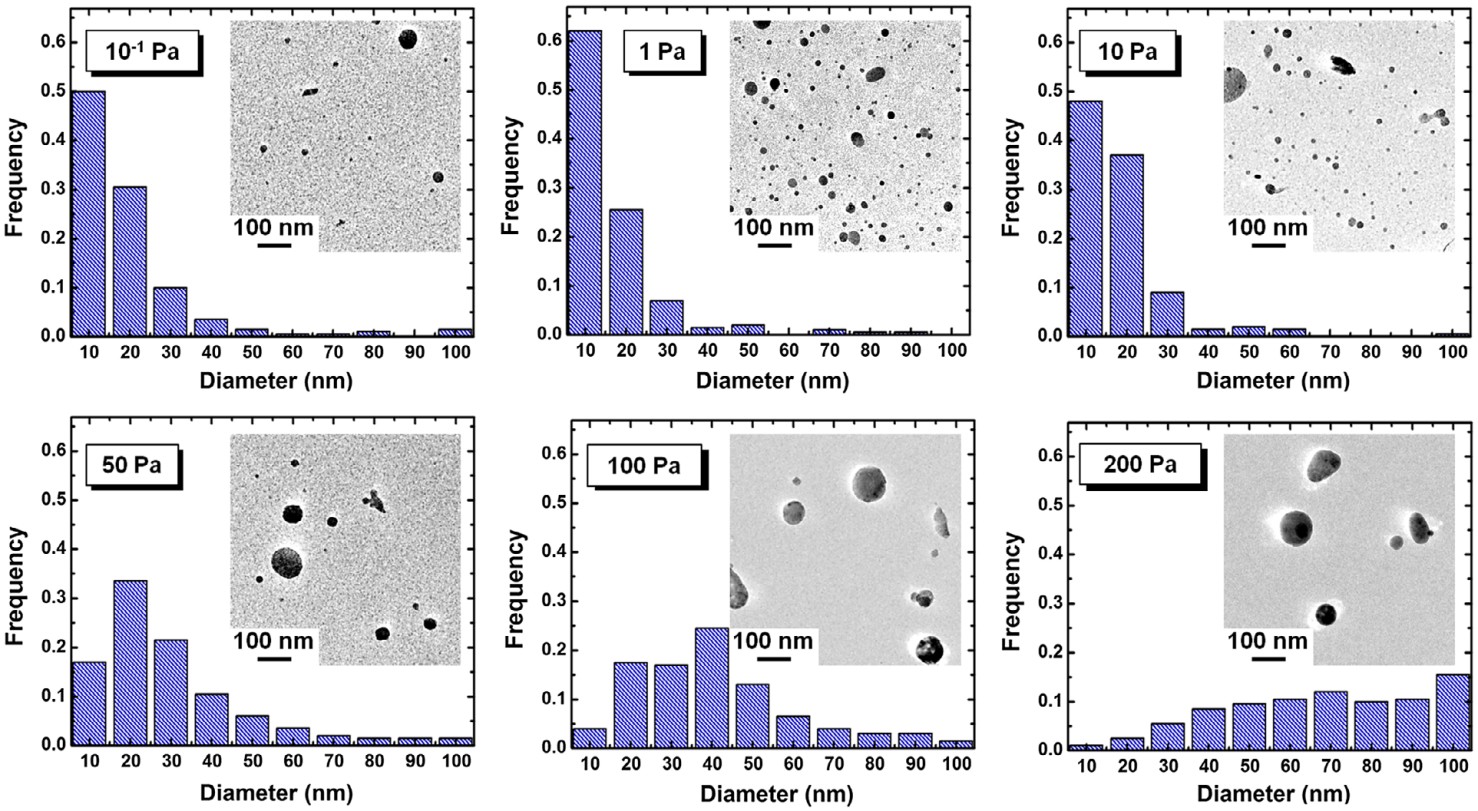
TEM images and size distributions of KMgF_3_ nanoparticles prepared under indicated gas pressure.

Figure 3 shows the TEM images and size distributions of KMgF_3_ nanoparticles prepared under indicated Ar pressures. Almost all the particles were spherical with diameters less than 100 nm and did not exhibit considerable agglomeration. Furthermore, almost all the particles with diameters <10 nm were single crystalline for all Ar pressures (Figure 2(a)). The observed particle shape may be influenced by surface tension during the nucleation and growth of the nanoparticle.[39–43] The size distributions were statistically analyzed using the TEM images by roughly measuring 300 nanoparticles. Since relatively large agglomerates with larger than submicron dimensions were occasionally mixed in with the nanoparticles, these larger particles were excluded from the measurements. The ratio of the volume of submicron particles to the volume of nanoparticles exceeded 10 for all Ar pressures except 0.1 Pa. Under an Ar pressure of 1 Pa, approximately 60% of the particles were less than 10 nm in diameter. In contrast, under higher Ar pressure, the peak of the size distribution histogram shifted toward larger diameter, and the peak width increased (Figure 3).

**Figure 4.**
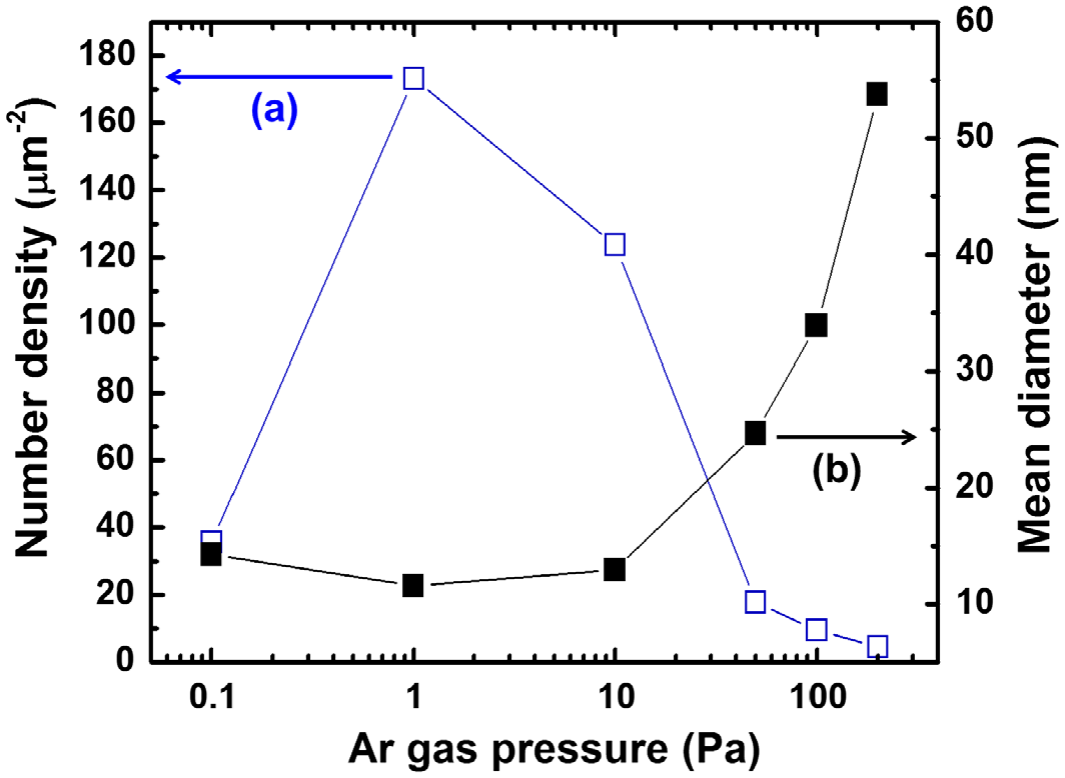
Dependences of (a) number density and (b) mean diameter on Ar gas pressure.

The mean particle diameters and number densities were also measured from TEM images (Figure 4). Under an Ar pressure of 1 Pa, we obtained the highest number density of 172.3 μm^−2^ and the smallest mean diameter of 12 nm. By increasing the Ar pressure to 200 Pa, the number density was dramatically decreased to 4.5 mm^−2^, and the mean diameter was increased to 54 nm. These results are attributed to the difference in collision frequency among ablated particles and Ar atoms. In the primary phase of gas-phase ablation, the collisions between ablated fine particles and ambient gas atoms play leading roles, resulting in the dissipation of their kinetic energy.[44,45] These effects determine the nucleation and the growth of nanoparticles. High Ar pressure caused frequent collisions, resulting in abundant growth. On the other hand, infrequent collisions resulted in low number densities at pressures under 10^−1^ Pa. The results suggest that an Ar pressure of 1 Pa is suitable for obtaining a large quantity of fine particles with diameters of several nm. In contrast to these dramatic changes caused by Ar pressure, the fluence and substrate arrangement had little effect on particle diameter in our experiments. For example, increasing the fluence from 7.6 to 16.2 J cm^–2^ changed the mean diameter from 12 nm (at 14.0 J cm^–2^) to 14 nm (at 7.6 J cm^–2^), while changing the substrate arrangement from *x* = 5 to 35 mm increased the mean diameter from 12 nm (at *x* = 5 mm) to 25 nm (at *x* = 35 mm).

During the above measurements, it was difficult to identify particles with diameters of less than 5 nm in the TEM images. We may be able to obtain a higher number density and a more detailed size distribution from the samples prepared under low Ar pressure by using a higher magnification or higher-resolution images.

**Figure 5.**
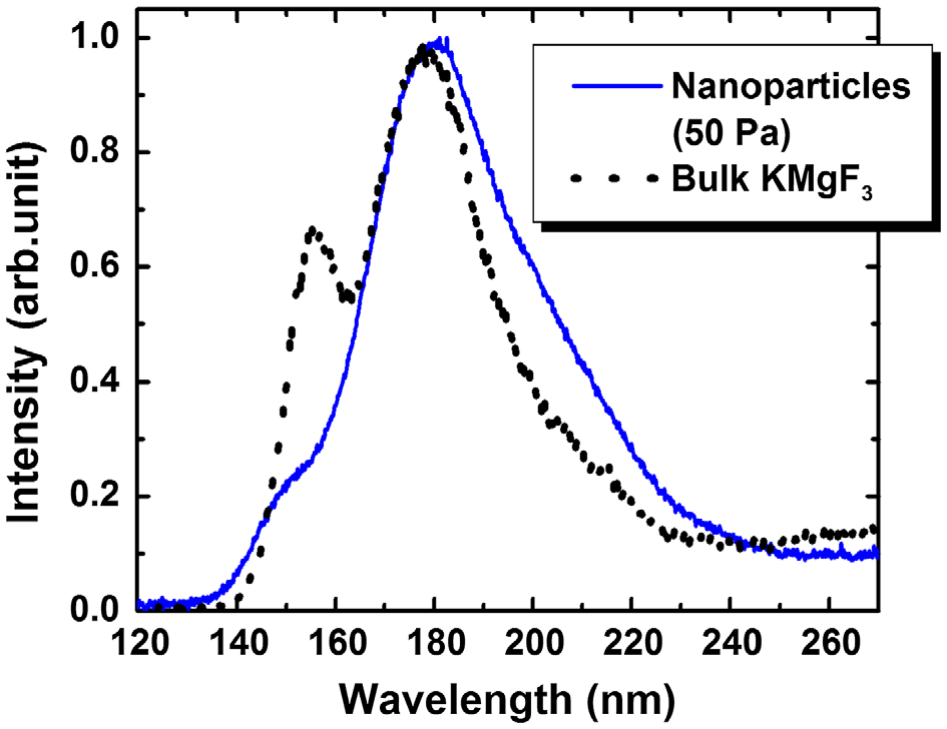
CL spectra of KMgF_3_ nanoparticles (blue solid line) and bulk KMgF_3_ (black dotted line).

Figure 5 shows the cathodoluminescence (CL) spectrum of KMgF_3_ nanoparticles prepared under an Ar pressure of 50 Pa; the CL spectrum of bulk KMgF_3_ is indicated by the black line in this figure. VUV fluorescence was observed under electron beam excitation in the wavelength region from 140 to 230 nm with a slight change in the spectrum shape. The main peak at 180 nm exhibited a long-wavelength tail. The peak at 150 nm became weak and shifted slightly toward shorter wavelengths. Since submicron-sized agglomerates occasionally became mixed with the nanoparticles in the method used in this study, it was difficult to exclude them from the CL measurements. Thus, we could not determine the relationship between particle size and CL spectrum. Note that the changes in spectral shape such as splitting have been reported in other materials;[46–48] however, this study represents the first such observation in the VUV region.

## 4. Conclusions

We have successfully fabricated size-controllable, nanosized KMgF_3_ single crystals via a dry PLA process with femtosecond laser pulses. Almost all the fabricated particles were spherical with diameters less than 100 nm, and the particles were not highly agglomerated. The crystal lattice fringes observed in the HR-TEM images and the spot patterns seen in the SAED patterns indicated that the particles are single crystals. The Ar ambient gas pressure influenced the particle size more strongly than other parameters such as fluence and substrate arrangement. At Ar pressures of approximately 1 and 10 Pa, relatively small particles were produced with high number densities. At higher gas pressures, the mean diameter increased, and the number density decreased dramatically. An Ar pressure of 1 Pa was suitable for obtaining a large quantity of fine particles with diameters of several nanometers in our experiments. Upon electron beam excitation, the KMgF_3_ nanoparticles emitted VUV fluorescence in the wavelength region from 140 to 230 nm with blue shift and broadening of spectrum. These results demonstrate the possibility of using KMgF_3_ nanoparticles as a novel VUV phosphor.

## Disclosure statement

No potential conflict of interest was reported by the authors.
